# Polymorphism of *Plasmodium falciparum *Na^+^/H^+ ^exchanger is indicative of a low in vitro quinine susceptibility in isolates from Viet Nam

**DOI:** 10.1186/1475-2875-10-164

**Published:** 2011-06-14

**Authors:** Véronique Sinou, Le Hong Quang, Stéphane Pelleau, Vu Nhu Huong, Nguyen Thu Huong, Le Minh Tai, Lionel Bertaux, Marc Desbordes, Christine Latour, Lai Quang Long, Nguyen Xuan Thanh, Daniel Parzy

**Affiliations:** 1 UMR-MD3 Relations Hôte-Parasite, Pharmacologie et Thérapeutique, Université de la Méditerranée, Institut de Médecine Tropicale du Service de Santé des Armées, Antenne IRBA- Marseille, Marseille, France; 2 Military Center of Preventive Medicine, Ho Chi Minh City, Vietnam; 3 Military Center of Drug Analysis and Research, Hanoi, Vietnam; 4 Military Institute of Hygiene and Epidemiology, Hanoi, Vietnam

## Abstract

**Background:**

The *Plasmodium falciparum *NA+/H+ exchanger (*pfnhe1*, gene PF13_0019) has recently been proposed to influence quinine (QN) susceptibility. However, its contribution to QN resistance seems to vary geographically depending on the genetic background of the parasites. Here, the role of this gene was investigated in *in vitro *QN susceptibility of isolates from Viet Nam.

**Method:**

Ninety-eight isolates were obtained from three different regions of the Binh Phuoc and Dak Nong bordering Cambodia provinces during 2006-2008. Among these, 79 were identified as monoclonal infection and were genotyped at the microsatellite *pfnhe1 *ms4760 locus and *in vitro *QN sensitivity data were obtained for 51 isolates. Parasite growth was assessed in the field using the HRP2 immunodetection assay.

**Results:**

Significant associations were found between polymorphisms at *pfnhe1 *microsatellite ms4760 and susceptibility to QN. Isolates with two or more DNNND exhibited much lower susceptibility to QN than those harbouring zero or one DNNND repeats (median IC_50 _of 682 nM *versus *median IC_50 _of 300 nM; *p *= 0.0146) while isolates with one NHNDNHNNDDD repeat presented significantly reduced QN susceptibility than those who had two (median IC_50 _of 704 nM *versus *median IC_50 _of 375 nM; p < 0.01). These QNR associated genotype features were mainly due to the over representation of profile 7 among isolates (76.5%). The majority of parasites had *pfcrt76T *and wild-type *pfmdr1 *(> 95%) thus preventing analysis of associations with these mutations. Interestingly, area with the highest median QN IC_50 _showed also the highest percentage of isolates carrying the *pfnhe1 *haplotype 7.

**Conclusions:**

The haplotype 7 which is the typical Asian profile is likely well-adapted to high drug pressure in this area and may constitute a good genetic marker to evaluate the dissemination of QNR in this part of the world.

## Background

Quinine (QN), an alkaloid from Cinchona Bark, is still a major anti-malarial drug especially for the treatment of severe or complicated malaria cases [1]. However, decreased sensitivity to QN has been reported, especially in South-East Asia [2-5], and the impact of this drug on malaria parasite genomes has been difficult to evaluate to date. There are some evidences suggesting that QN resistance is a multifactorial mechanism, which includes at least mutations in transporter genes *pfcrt *(*Plasmodium falciparum *chloroquine resistance transporter) and *pfmdr1 *(*P. falciparum *multidrug resistance 1) [6-14]. *In vitro *genetic and physiological studies have suggested that the *P. falciparum *sodium/hydrogen exchanger (*pfnhe*1) gene (PF13_0019) might also contribute to QN resistance [8,11,14]. Studies carried out on *P. falciparum *strains from several parts of the world provide evidence of significantly reduced QN activity in parasites harbouring two or more DNNND repeated motif in the coding microsatellite ms4760 of *pfnhe1 *[8,15]. Some of them further supported an association between decreased number of another repeated motif NHNDNHNNDDD and increased IC_50 _to QN [15]. In a series of 60 culture-adapted isolates from the China-Myanmar border, the involvement of DNNND and NHNDNHNNDDD in reduced QN susceptibility was confirmed [16]. However, these results were conflicting with those obtained with African isolates. Some studies carried out on field isolates from the Kenyan cost [17] and Uganda [18] have shown that reduced QN susceptibility was associated with two DNNND repeats combined with *pfmdr1 *mutations, whereas the presence of 1 versus 3 and 2 versus 3 DNNND repeats was associated with the restoration of QN susceptibility. In the other hand, no reliable association between *pfnhe1 *polymorphisms and *in vitro *QN response was observed in isolates from various African countries [19,20]. In Africa, QN treatment is still effective and QN resistance is much less frequently found than in Asia. As recently reviewed in Okombo *et al *[21] African isolates show lower IC_50 _values (20 to 600 nM), while in South-East Asia, IC_50 _can reach values above 1200 nM. Thus, to validate *pfnhe1 *as a genetic marker of QN resistance in the field, more studies using samples from South East Asia with reduced susceptibility to QN are needed. Moreover, some of these studies included isolates collected from disparate regions with different drug history and over a longer period. Also, evaluation of parasites after culture adaptation may include a bias in the selected genotypes as different parasite populations in the same sample do not thrive equally well in *in vitro *culture [22,23]. In this work, the association between *pfnhe1 *polymorphism and *in vitro *QN susceptibility of fresh clinical *P. falciparum *isolates collected from a single geographical region along the Viet Nam-Cambodia border was investigated. *In vitro *tests were conducted using an mobile laboratory set up in the field to optimize the time gap between the collection of blood samples on patients and the culturing of these samples.

## Methods

All culture and ELISA procedures were performed at a small temporary field laboratory set up at the dispensary of Bom Bo in Binh Phuoc province. Blood samples were transported at +4°C within 12 hours of collection. Samples for PCR genotyping were frozen at -20°C until shipment to the UMR-MD3 laboratory, Marseille, France.

### Study site

**Figure 1:**
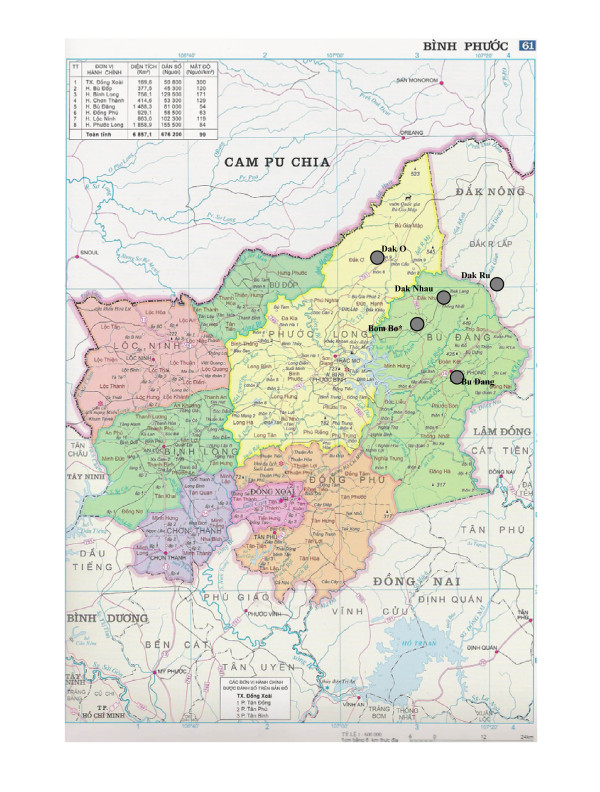
**Map of malaria monitoring sites in Phuoc Long and Bu dang districts (Binh Phuoc province) and Dak R'Lap district (Dak Nong province), Vietnam**. * The mobile laboratory was set up at the dispensary in Bom Bo.

The study was conducted in two neighbouring regions bordering Cambodia: the Binh Phuoc province in the South-East and the Dak Nong province in the Centre of Viet Nam (Figure 1). To ensure sufficient sample recruitment within the timeframe in view of the low population density and transportation difficulties, four sites were chosen in 20-25 km radius of the clinic of Bom Bo. All are situated in hilly and forested parts of Binh Phuoc and Dak Nong provinces. These areas are highly endemic and it is a region where multidrug resistance to anti-malarials has spread [1,24].

### Patients and clinical samples

Isolates were obtained from patients attending in commune health centres for uncomplicated malaria by collecting venous blood sample (7 ml) in Vacutainer^Ò ^tubes coated with EDTA (Vacutainer tubes, Becton Dickinson, Rutherford, NJ). Malaria positivity was evaluated by using rapid diagnostic based on the detection of Plasmodium-specific lactate dehydrogenase (pLDH) (OptiMAL-IT, DiaMed AG, Switzerland). Patients gave their consent for this study and were questionned about socio-demographic data and drug intake. The study was approved by the Viet Nam People's Army Department of Military Medicine. Positive patients were treated with dihydroartemisinin-piperaquine combination in accordance with the national drug policy of Viet Nam.

### Mobile laboratory

The mobile laboratory was send to Ho Chi Minh City (Viet Nam) from Marseille (France) using a commercial flight, and then transported to the clinic of Bom Bo (about 130 km north from HCMC) on a pick-up by the Vietnamese army. It was constituted by four compartimented boxes (350 kg; 2 m^3^) outfitted for the assessment of *P. falciparum *susceptibility to anti-malarials. These boxes, which composed the lab environment, contained a mini-class II biological safety cabinet, an incubator chamber equipped with N_2 _and CO_2 _gaz bottles (5 L steel cylinders), a microscope, a centrifuge, a field-suitable ELISA plate washer (Hydroflex Platform, Tecan, Austria) and plate reader (Sunrise Absorbance Reader, Tecan, Austria). It also contained a generator (SHX1000, Elemax, Japan) and an inverter to protect all the electrical apparatus. The lab unit was completed by a cold chain to keep *in vitro *microtest plates and ELISA reagents at +4°C, and culture reagents at - 20°C during the transport. Once on site, the mobile laboratory was placed in a covered place. Mains electricity was used, and a generator in case of power cut. A refrigerator and a deep freeze were installed for the storage of reagents.

### *In vitro *drug assay

*In vitro *microtest plates were prepared at UMR-MD3, Marseille, France and the quality controls were performed with the QN-sensitive 3D7 (unknown origin, MR4-ATCC) and QN-resistant K1 (Thailand, MR4-ATCC) and Dd2 (Indochina, MR4-ATCC) strains using the isotopic microtest method [25]. Final drug concentrations ranged from 12.5 to 3200 nM.

In the field, blood samples were washed once with parasite culture medium and immediately transferred to *in vitro *pre-dosed drug plates as described previously [26]. The plates were incubated at 37°C in presence of 5% CO_2_, 85% N_2 _and 10% O_2 _for 72 h. After culture the plates were frozen down at -20°C. Parasite growth inhibition was quantified using a commercial HRP2 ELISA kit (Malaria Ag CELISA; Cellabs, Australia) according to the manufacturer's specifications. Spectrophotometric analysis was performed using a small, field-suitable ELISA plate reader (Tecan Sunrise Absorbance Reader, Tecan Austria, Austria) at an absorbance of 450 nm.

#### Molecular analysis

Parasite DNA was extracted from blood samples using QIAamp DNA minikit (Qiagen, Germany).

Parasite samples were genotyped at six microsatellites loci Pf2689 (chr. 12, Genbank ID G37854), Pf2802 (chr. 5, G37818), C4M69 (chr. 4, Genbank ID G37956), C4M79 (chr. 3, Genbank ID G42726), TRAP (chr. 13, Genbank ID G37858) and 7A11 (chr. 7, Genbank ID G38831) using previously described method [27].

PCR conditions used to detect polymorphism in *pfcrt *at codon 76 and *pfmdr1 *at codons 86, 184, 1034, 1042 and 1246 were as described elsewhere [28]. *Pfnhe1 *was amplified by PCR using a reaction volume of 25 μL containing 2.5 μL of sample DNA, 0.3 μM of each primer (NHE-F: 5'-AATCCCTGTTGATATATCG-3' and NHE-R: 5'-GTCTTGCAGTGCATGGACC-3'), 200 μM of dNTPs, 4 mM MgCl_2_, 1 U GoldStar DNA polymerase (Eurogentec, Seraing, Belgium), PCR buffer 10 × (750 mM Tris-HCl pH 8.8, 200 mM (NH_4_)_2_SO_4_, 0.1% (v/v) Tween 20 and stabilizer) (Eurogentec, Seraing, Belgium). The PCR assay was performed for 40 cycles at 94°C for 20 sec, 50°C for 20 sec and 72°C for 40 sec. The denaturation and final extension were carried out at 94°C for 5 min and at 72°C for 5 min, respectively. PCR products were purified using High Pure PCR Product Purification Kit (Roche Diagnostics, Meylan, France) and sequenced with the ABI PRISM Big Dye Terminator v3.1 Cycle Sequencing Kit (Applied Biosystems, Foster City, CA, USA). Fluorescent sequence products were analysed by an ABI PRISM 3100 Genetic Analyzer (Applied Biosystems).

### Statistical analysis

Drug concentrations inhibiting parasite growth by 50% (IC_50_) were calculated using nonlinear regression analysis of log-based dose-response curves (Riasmart; Packard). Mann-Whitney U-test was used to compare median IC_50 _between two groups and Kruskal Wallis in case of multiple groups. Fisher's exact test was used for comparison of frequencies between groups when categorical variables were defined. All analysis was done with GraphPad Prism 5.0.

### Nucleotide sequence accession numbers

Nucleotide sequence of new ms4760 genotypes were deposited in the GenBank database under accession numbers GQ845118, GQ845119 and GQ465284.

## Results and discussion

A total of 98 clinical *P. falciparum *isolates were collected between 2006 and 2008. All isolates were genotyped using six microsatellites loci (Pf2689, Pf2802, C4M69, C4M79, TRAP and 7A11) to exclude polyclonal infections, leaving 79 monoclonal isolates. Ten different *pfnhe1 *ms4760 microsatellites profiles were found in these 79 isolates (Table 1), including three not previously described (ms4760-63, -64, and -65). The most prevalent profile was ms4760-7, found in 68.3% (*n *= 54/79) of the isolates, followed by profiles ms4760-1 (7.6%, *n *= 6/79), ms4760-6 (7.6%, *n *= 6/79) and ms4760-3 (6.3%, *n *= 5). The number of DNNND repeats in the 10 *pfnhe1 *profiles varied from zero to four, and three repeats were more common accounting for 72.1% (n = 57/79) of the isolates. In addition, the majority of isolates (83.5%; *n *= 66/79) had a single repeat of NHNDNHNNDDD while the remaining had two repeats (16.4%; *n *= 13/79). The overrepresentation of parasite isolates having three DNNND and one NHNDNHNNDDD repeat was due to the high frequency of the profile 7 among isolates. This observation is in line with previous studies in which the haplotype 7 was found to be predominant in samples from Asia [8,15,16]. Parasites with this most abundant ms4760 haplotype were associated with increased QN IC_50 _[15,16]. Then, the reduced genetic diversity of *Pfnhe1 *profiles towards three DNNND repeats in our study would be suggestive of a low level QN responsiveness.

**Table 1:** Sequence polymorphism and distribution of the 10 *Pfnhe-1 *ms4760 haplotypes observed in the 79 Vietnamese parasite isolates

**No. Genotype profile**	**Sequence**									**N (freq.)**
	**DNNND repeat**					**NHNDNHNNDDD repeat**				
ms-1:	KKKKISGSNNDNN	**DNNND**	**DNNND**			NHNDDKNNKNDDD	**NHNDNHNNDDD**	**NHNDNHNNDDD**	NNNDNHNDDDNNSSHY	6(7.6)
ms-3:	KKKKISGSNNDNN	**DNNND**				DKNNKNDDD	**NHNDNHNNDDD**	**NHNDNHNNDDD**	NNNDNHNDDDNNSSHY	5(6.3)
ms-5:	KKKKISGSNNDNN	**DNNND**	**DNNND**	**DNNND**	**DNNND**	NHNDDKNNKNDDD	**NHNDNHNNDDD**		NNDNHNDDDNNSSHY	1(1.3)
ms-6:	KKKKISGSNNDNN	**DNNND**	**DNNND**			NHNDDKNNKNDDD	**NHNDNHNNDDD**		NNNDNHNDDDNNSSHY	6(7.6)
ms-7:	KKKKISGSNNDNN	**DNNND**	**DNNND**	**DNNND**		NHNDDKNNKNDDD	**NHNDNHNNDDD**		NNNDNHNDDDNNSSHY	54(68.3)
ms-18:	KKKKKSGSNNDNN	**DNNND**	**DNNND**			NHNDDKNNKNDDD	**NHNDNHNNDDD**	**NHNDNHNNDDD**	NNNDNHNDDDNNSSHY	2(2.5)
ms-49:	KKKKISGSNNDNN	**DNNND**	**DNNND**	**DNNND**		NHNDDKNN**N**NDDD	**NHNDNHNNDDD**		NNNDNHNDDDNNSSHY	1(1.3)
ms-63:	KKKKISGSNNDSN	**DNNND**	**DNNND**	**DNNND**	**DNNND**	NHNDDKNNKNDDD	**NHNDNHNNDDD**		NNNDNHNDDDNNSSHY	1(1.3)
ms-64:	KKKKISGSNNDNN	**DNNND**	**DNNND**	NNN**N**	D	NHNDDKNNKNDDD	**NHNDNHNNDDD**		NNNDNHNDDDNNSSHY	2(2.5)
ms-65:	KKKKISGSNNDNN	D				NHNDDKNNKNDDD	**NHNDNHNNDDD**	**NHNDNHNNDDD**	NNNDNHNDDDNNSSHY	1(1.3)

Underlined letters indicate mutated amino acids. In brackets, frequency (freq.) of the genotype profile.

In the absence of clear clinical resistance, strict cut-off level for *in vitro *QN resistance has not been yet defined. Then, different arbitrary cut-off points of 800 nM, 500 nM or 300 nM have been proposed depending on the origin of isolates and the methodology used [29]. In this study, methodologies used for the parasite culture and to assay drug response were standardized to be used in the reference laboratory and in the field. The QN susceptibility of three strains (3D7, Dd2 and K1) that display different QN sensitivity was measured by the HRP2 ELISA assay using the same batches of pre-dosed drug plates than that used in the field. The IC_50 _values for 3D7, K1 and Dd2 were 126.3 ± 0.4 nM, 825.0 ± 76.8 nM and 1192.0 ± 74.3 nM, respectively. These results were comparable with those obtained using the gold standard isotopic assay (127.1 ± 12.5 nM for 3D7, 857.4 ± 31.5 nM for K1 and 1148.8 ± 111.7 nM for Dd2) and were consistent with the separation of the 3D7 and Dd2 strains respectively into the QNS and QNR categories and with the intermediate QN susceptibility of the K1 strain. Then, the QN cut-off value in this study was set at 800 nM.

Fifty-one monoclonal isolates were successfully evaluated for *in vitro *susceptibilities to QN. The IC_50 _median [25-75% interquartiles] *in vitro *activity of QN was 650 nM [517-928]. A substantial proportion of the isolates (35.3%, *n *= 20) showed reduced QN sensitivity with IC_50 _above 800 nM (median of 972.9 nM [914.2-1028.8 nM]). Isolates originating from another hyper endemic part of Southeast Asia and with high QN IC_50 _values were also recently reported [16]. The observation of a large proportion of isolates from South central Viet Nam with harmful decreased QN responsiveness is in line with a clinical study conducted in the neighbouring Lam Dong province, in which reduced sensitivity to QN expressed as a lower rate of parasite clearance was observed [5]. The decreasing sensitivity to QN observed here suggested that isolates have been exposed to a strong QN pressure in this part of Viet Nam. In Viet Nam, QN has been consistently used alone or in combination until 2006 and is now reserved as a second-line treatment of uncomplicated and severe falciparum malaria [30]. However, when the study was conducted, QN was still available in local shops (personnal data); thus its use as self-medication would be a factor in the persistence of QN resistance especially in rural and remote areas, which have limited basic health care facilities [31-33].

**Figure 2:**
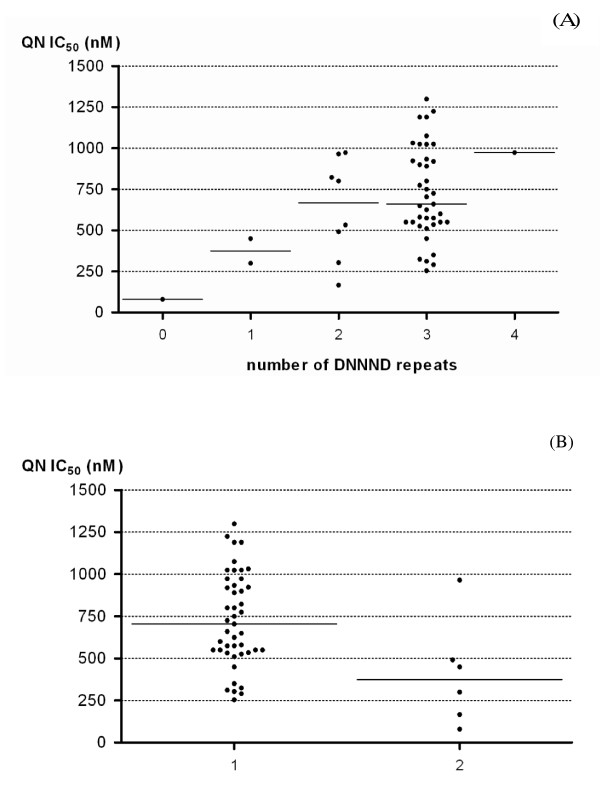
**Association between QN IC_50 _(nM) of parasite isolates and the number of (A) DNNND repeats or (B) NHNDNHNNDDD repeats in *Pfnhe-1 ***. (A) Median QN IC_50 _of isolates with 2 or more DNNND repeats are significantly higher than isolates with less than 2 DNNND (p = 0.015) (B) Median QN IC_50 _of isolates with 1 NHNDNHNNDDD repeat are significantly higher than isolates harbouring 2 repeats (p < 0.01).

Out of the 51 isolates that were successfully tested for drug susceptibility, seven *pfnhe1 *ms4760 profiles were detected. The distribution of *pfnhe1 *profiles was not statistically different than that found in the whole set of parasites (*n *= 79). The *pfnhe1 *profile 7 was still predominant, accounting for 76.5% (*n *= 39/51), followed by haplotypes 6 (9.8%, *n *= 5/51), 1 (3.9%, *n *= 2/51) and 3 (3.9%, *n *= 2/51). The other ms4760 haplotypes (profiles 5, 18 and 65) were found only in one isolate each (2%, *n *= 1/51). Significant associations were found between *pfnhe1 *polymorphisms and susceptibility to QN. Isolates with two or more DNNND were less susceptible to QN than those harbouring zero or one DNNND repeats: median QN IC_50 _= 682 nM [535-941] *vs*. median QN IC_50 _= 300 nM [190-375] (Figure 2A; p = 0.0146), respectively. In addition, isolates having one NHNDNHNNDDD repeat presented significantly reduced QN susceptibility (median QN IC_50_= 704 nM [550-933]) than those who had two (median QN IC_50_= 375 nM [200-482]) (Figure 2B; p < 0.01). These results are in accordance with previous studies performed with culture-adapted isolates which reported a link between the number of DNNND repeat and QN responsiveness [8,15,16], but this is the first time that this association is reported on freshly collected clinical isolates. In addition, it should be mentioned that the majority of our parasites isolates contained the *pfcrt *CQR associated genotype (84%) and wild-type *pfmdr1 *(> 95%) thus preventing analysis of associations with these mutations.

Given the multifactorial basis of QNR and the strain-specific contribution of *pfnhe1 *to QN phenotype [7,14,33-36], it may be suggested that the association between *pfnhe1 *and QN responses in our study may has been rendered possible due to a reduced genetic diversity, highlighed by the high frequency of the likely well-adapted *pfnhe1 *variant, namely ms4760-7. Indeed, this profile with three DNNND repeats is currently found with high frequency among Asian parasites [8,15,16]. In particular, when isolates were collected from a relatively small geographic region with the same drug use history the frequency of haplotype 7 accounted for almost 50% [16] up to 68% in our study. If this genotype is actually linked to the phenotype of a decrease susceptibility to QN with a high penetrance, its overrepresentation in Asian isolates may explain why the association of *pfnhe1 *polymorphism with QN susceptibility has always been observed, whatever the methodology used, i.e. use of culture-adapted parasite isolates [16] or freshly collected clinical isolates [this study]. All together, this would suggest that the prevalence of profile 7 may constitute a good marker of QNR spread at least in Viet Nam and likely in South-East Asia.

To complete this hypothesis of a relationship between QN susceptibility and the frequency of haplotype 7, samples were further examined according to the area where they were collected. Among isolates with QN IC_50_, 32 were from Bu Dang, 12 were from Phuoc Long and 7 were from Dak R'Lap district. QN susceptibilities or frequencies of isolates with reduced QN susceptibility were statistically different between the three districts (Table 2). The Phuoc Long district, which presented the highest median QN IC_50_, with half of the isolates having the highest values of IC_50 _(> 800 nM), had also the highest percentage of isolates carrying the *pfnhe1 *haplotype 7 (93.7%). Although concerning less isolates, the Dak R'Lap district presented the lowest frequency of isolates with reduced susceptibility to QN (28.6%) and the lowest frequency of profile 7 (46.7%) compared to other districts (p < 0.01). Finally the Bu Dang district, with an intermediate frequency of 37.5% of isolates having QN IC_50 _> 800 nM had also an intermediate level of profile 7 frequency (72.9%). This appears to agree with the malaria epidemiology in these areas where a higher number of cases of malaria has been reported in Phuoc Long compared to Dak R' Lap and Bu Dang [33]. In this study, the monitoring site in Phuoc Long was located in a rural and remote forested region and was 10 km far from border with Cambodia, where there is significant population movement [37]. The neighbouring Kratie and Modulkiri Cambodian provinces are characterized by intense malaria transmission [38] and multidrug resistant *P. falciparum *[39]. Consequently, the high proportion of QNR found among isolates from Phuoc Long may either be due to inherently QN-resistant parasite population or to possible spread of resistant isolates from the neighbouring Cambodia. In particular, migrant workers have been suspected as a leading cause of malaria transmission in these areas as they are at particularly high risk of malaria and may have poor access to preventive and therapeutic services [40]. Then, in the presence of substantial population movement, high transmission rate and the long terminal half-life QN, selection of resistant strains may be accelerated in this area.

**Table 2:** QN susceptibility and frequency of *pfnhe-1 *haplotype 7 among the three geographical districts

District	Median QN IC_50 _(nM) [interquartile range]	Percentage of QNR isolates (*n/N*)	Percentage of haplotype 7 (*n_1_/N_1_*)
Dak R'Lap	535[418-849]	28.6 (2/7)	46.7 (7/15)*
Bu Dang	618[450-906]	37.5 (12/32)	72.9 (34/48)
Phuoc Long	770[595-986]	50.0 (6/12)	93.7 (15/16)

1. n = number of QNR parasite isolates; N = number of parasite isolates with QN IC_50_.

2. n_1 _= total number of parasite isolates carrying the haplotype 7; N_1 _= number of parasite isolates genotyped for *pfnhe-1*.

* The percentage of profile 7 is lower in isolates from Dak R'Lap district than that of isolates from Bu Dang or Phuoc Long districts (p < 0.01).

According to the present study and the previous study of Meng *et al *[16], the typical Asian haplotype 7 appears likely well-adapted to high drug pressure areas, and the local correlation we found between its frequency and the frequency of isolates with reduced susceptibility to QN would suggest that it may constitute a good genetic marker to evaluate the dissemination of QNR in this part of the world.

In conclusion, the mechanism of QN resistance is complex with polymorphisms in multiple genes contributing to the phenotype. However, to date no single mutation of set of polymorphisms has been shown to be a robust marker for *in vitro *QN sensitivity of field isolates. In the present study, the frequency of haplotype 7 was the most relevant but additional markers are needed to define parasite as QNR or QNS. Further studies are needed to confirm whether the haplotype 7 is necessary for the emergence QN resistance and can be used in monitoring the QNR spread in South East Asia.

## Competing interests

The authors declare that they have no competing interests.

## Authors' contributions

NXT, LQL, LHQ and DP planed the field study; VS, DP, MD, LMT, VNH and NTH collected samples; VS, DP, MD, NTH accomplished all *in vitro*-susceptibility testing on the field. CL did *in vitro *assays at the laboratory, Marseille, France. LB conducted molecular analysis. SP performed the statistical analysis and interpretation of the results. VS drafted the manuscript. SP, LB and DP revised the manuscript. All authors read and approved the final version.
